# Perioperative Management of a Toddler With Tuberous Sclerosis and Dilated Cardiomyopathy Undergoing Open Abdominal Aortic Aneurysm Repair: A Case Report

**DOI:** 10.1155/cria/9513720

**Published:** 2026-10-16

**Authors:** Monica Ho, Jason Brown, James Duca

**Affiliations:** ^1^ John A. Burns School of Medicine University of Hawaiʻi 651 Ilalo Street Honolulu Hawaii 96813 USA; ^2^ Department of Anesthesiology at Kapiʻolani Medical Center and Hawaiʻi Pacific Health Medical Group 1319 Punahou St Honolulu Hawaii 96826 USA

**Keywords:** abdominal aortic aneurysm, cardiomyopathy, child, dilated, echocardiography, pediatric anesthesia, preschool, transesophageal, tuberous sclerosis

## Abstract

Toddler abdominal aortic aneurysms (AAA) are rare and lack defined perioperative guidelines. Aneurysms may be associated with tuberous sclerosis complex (TSC) but rarely occur with concurrent dilated cardiomyopathy. This case report describes the anesthetic and surgical considerations for a two‐year‐old male with TSC and dilated cardiomyopathy with low ejection fraction undergoing open infrarenal AAA repair. Our most important perioperative considerations included advanced hemodynamic monitoring, management of intraoperative blood loss, and the physiological changes associated with aortic clamping. Transesophageal echocardiography was used throughout the operation to guide inotropic support and assess ventricular filling using renal‐protective strategies and balanced transfusion.

## 1. Introduction

Fewer than 30 cases of pediatric abdominal aortic aneurysms (AAA) have been reported, with the majority occurring in association with congenital connective tissue disorders or vasculitides [1]. There is a known association of AAA patients with tuberous sclerosis complex (TSC) in varying age groups, though it is rare in the pediatric population [2]. Vascular abnormalities in TSC are thought to result from dysregulated smooth muscle proliferation caused by mTOR pathway activation, leading to medial degeneration and aneurysm formation [3]. Dilated cardiomyopathy is an extremely rare manifestation in TSC but has been linked to arrhythmias from cardiac rhabdomyomas [4].

There is no universal management for pediatric AAAs. Conservative management has demonstrated a significantly high mortality rate due to rupture, cardiac dysfunction, or renal failure [5]. Early surgical intervention is recommended; however, surgical repair of AAA with concurrent dilated cardiomyopathy poses significant challenges due to the balance between preload dependency and volume overload risk [6]. Although rarely used in pediatric AAA repair, continuous transesophageal echocardiography (TEE) has emerged as a useful tool in high‐risk noncardiac procedures, especially in patients with cardiomyopathy [7].

This case report discusses TEE‐guided hemodynamic management of dilated cardiomyopathy with reduced ejection fraction during pediatric open AAA repair. Our most important perioperative considerations included advanced hemodynamic monitoring, management of intraoperative blood loss, and the physiological changes associated with aortic clamping.

## 2. Case Presentation

The patient was a 12.1‐kg two‐year‐old male traveling from a Pacific Island nation with a history of TSC. At 3 months of age, he developed seizures characterized by eye rolling and symmetric body stiffening that were poorly controlled despite escalating doses of levetiracetam. Evaluation in New Zealand revealed subcortical tubers, a subependymal giant cell astrocytoma (SEGA), and right renal hypoplasia with multiple bilateral renal cysts, consistent with TSC. Due to progressive seizures, his family traveled to Hawaii for further management. On arrival, he developed a new‐onset generalized tonic‐clonic seizure, tactile fever, and nasal congestion and presented to the emergency department.

On admission, vital signs were as follows: temperature 36.8°C (98.2°F), pulse 124 beats/min, blood pressure 110/64 mmHg, respiratory rate 28 breaths/min, and SpO_2_ 98%. Physical examination revealed two ash leaf spots and generalized hypotonia with an otherwise normal neurological examination. Infectious workup was negative, and upper respiratory symptoms were most consistent with a viral infection given similar symptoms in family members. EEG revealed generalized slow spike‐wave activity at 2 Hz, multifocal sharp waves in the parietotemporal regions, and clusters of tonic seizures. These findings were compatible with epileptic encephalopathy, suspected to be Lennox–Gastaut syndrome. He was started on vigabatrin, which was gradually increased every 3 days, reaching 75 mg/kg twice daily, in addition to his baseline levetiracetam. Seizures remained controlled throughout hospitalization.

A retroperitoneal ultrasound was performed to evaluate his TSC‐related renal involvement further and incidentally revealed an unruptured AAA measuring 4 cm. On follow‐up CT angiogram, the aneurysm measured 4.3 × 3.8 cm and extended from the infrarenal aorta to the bifurcation, which was a new finding compared with prior imaging from 1 year ago.

Preoperative EKG was unremarkable. Initial transthoracic echocardiogram (TTE) revealed a moderately dilated left ventricle (LV) without hypertrophy, global hypokinesis, a patent foramen ovale with left‐to‐right shunting, and a biplane ejection fraction of 33.3% (Figure 1). Valvular function and right ventricular morphology and function were normal. No cardiac rhabdomyomas were seen. Cardiac markers were also elevated, with a proBNP of 6456 pg/mL and troponin 50 ng/L. These findings were consistent with dilated cardiomyopathy; however, it remained unclear if this was due to an acute vs. chronic process. Viral myocarditis could not be ruled out given his fever and upper respiratory symptoms. No prior ECHOs were available for comparison. To reduce the risk of volume overload, furosemide was started. Afterload reduction with enalapril was considered but ultimately held in light of the AAA. Given the unknown acuity of his AAA and risk for spontaneous rupture, he was transferred to PICU for higher‐level monitoring.

**FIGURE 1:**
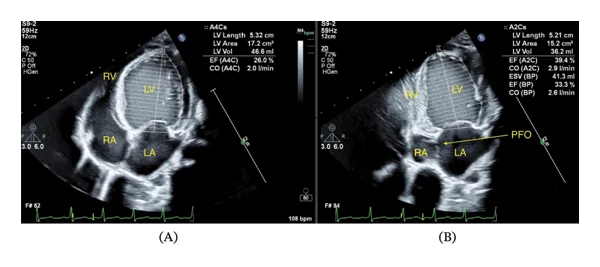
Initial transthoracic echocardiogram in the apical four‐chamber (A) and two‐chamber (B) views with automated Simpson’s biplane endocardial tracing (BP) of 33.3%. This study revealed an enlarged left ventricle (LV) measuring 17.2 cm^2^ in the four‐chamber view and global hypokinesis. A patent foramen ovale (PFO) is visible with left‐to‐right shunting.

Due to the size of the AAA, risk of rupture, and cardiomyopathy, surgical intervention was recommended during this hospitalization. The rarity of this case required multidisciplinary expertise in the operating room, including an adult vascular surgery team, pediatric surgery team, a pediatric cardiologist, and two pediatric anesthesiologists trained in TEE and congenital heart cases. After a thorough discussion, the team proceeded with an open repair approach using a polytetrafluoroethylene (PTFE) graft.

### 2.1. Intraoperative Management

Anesthesia induction was carefully initiated via sevoflurane, midazolam 1.5 mg, fentanyl 25 mcg, ketamine 20 mg, and rocuronium 10 mg through a peripheral catheter. Sevoflurane was used for maintenance and fentanyl for analgesia. Due to the risk of massive blood loss, transfusion was guided by serial point‐of‐care hematologic (hemoglobin/hematocrit and coagulation panels) and metabolic measurements (arterial blood gas and lactate), intraoperative blood loss, hemodynamic assessment, and TEE. It was achieved with packed red blood cells (PRBCs), fresh frozen plasma (FFP), platelets, and cell salvage. Calcium chloride was administered to augment contractility and correct hypocalcemia during transfusion. The patient’s scheduled antiepileptic medications were also continued perioperatively.

Monitoring included an arterial catheter, central venous pressure (CVP), urine catheter, and continuous TEE. TEE provided real‐time assessment of preload and contractility during cross‐clamping and reperfusion. Initial mid‐esophageal four‐chamber view revealed a markedly dilated LV with moderately depressed systolic function and an estimated EF of 30%–35%. Infusions of milrinone at 0.5 mcg/kg/min and epinephrine at 0.05 mcg/kg/min were initiated early and titrated by the TEE findings.

Approximately 5–7 mm of healthy aorta was present proximal to the aneurysm, allowing for infrarenal clamping. Before clamping, blood pressure was reduced by limiting intravenous fluids and increasing sevoflurane. Mannitol was given for osmotic diuresis and renal perfusion, and the lower‐body warming device was turned off to slow the metabolic rate. Heparin 100 units/kg was administered over 10 min, with an additional bolus given just before clamping to achieve an activated clotting time of 200–220 s. After aortic and iliac cross‐clamping, CVP decreased from 8 to < 5 mmHg (Figure 2). TEE demonstrated persistent LV dilation (Figure 3) with diminishing contractility under the increased afterload, prompting increases in milrinone to 0.75 mcg/kg/min and epinephrine to 0.10 mcg/kg/min. A restrictive fluid intake was maintained, as LV filling appeared stable.

**FIGURE 2:**
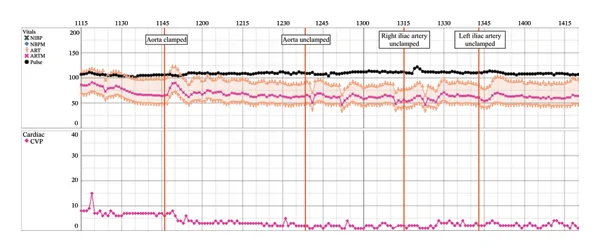
Hemodynamic effects of cross‐clamping and unclamping. Mean arterial pressure (MAP), noted by ARTM, transiently increased after aortic clamping before returning to baseline, while central venous pressure (CVP) progressively decreased during the clamp period. After aortic unclamping, MAP decreased with minimal change in heart rate and remained relatively stable with the gradual release of the right and left iliac artery clamps, inotropic support, and transfusion.

**FIGURE 3:**
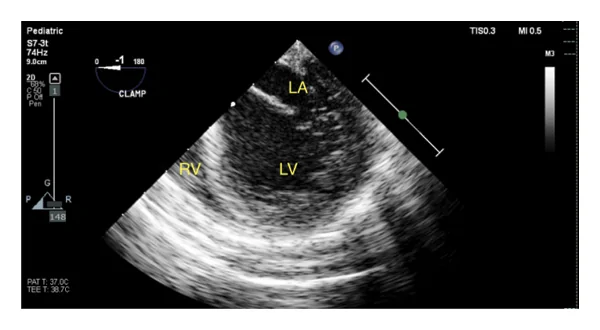
Intraoperative transesophageal echocardiogram in a modified mid‐esophageal four‐chamber view during cross‐clamping, demonstrating persistent left ventricle (LV) dilation.

A 12 × 6 mm bifurcated PTFE graft was used to replace the diseased aorta with intentional redundancy to accommodate future growth. Prior to unclamping the aorta, preload was normalized and inotropic support was increased to mitigate hypotension and myocardial depression. An additional bolus of mannitol and 1200 units of heparin were also administered. Total proximal aortic cross‐clamp time was 49 min. During reperfusion, i‐STAT testing revealed mild hyperglycemia and hyperkalemia, which were corrected with 0.5 units of insulin.

The aneurysm sac was dissected, revealing a large clot in the posterior wall of the infrarenal aortic neck that was sent for pathologic evaluation because of inflammatory features. End‐to‐end anastomosis was achieved with the right common iliac artery, and flow was restored to the right leg. The left common iliac artery was completely occluded. Papaverine was injected into both iliac arteries to reduce the risk of spasm; it was selected over glyceryl trinitrate because its local effect was expected to produce less systemic vasodilation.

Before iliac unclamping, norepinephrine was initiated at 0.05–0.1 mcg/kg/min in anticipation of hypotension. MAP transiently decreased to 50 mmHg after unclamping, with minimal changes to heart rate and no sustained hypotension. TEE demonstrated stable LV filling and systolic function, suggesting that the patient tolerated unclamping without clinically significant myocardial decompensation. Doppler revealed strong signals of bilateral femoral pulses. 80%–90% of the graft was covered by closing the aneurysm sac edges over it, except for the proximal 2 cm near the graft neck, which required omental coverage. Heparin was then reversed with protamine.

Lactate increased from 1.63 mmol/L at baseline to 3.62 mmol/L after left iliac unclamping and peaked at 4.81 mmol/L 1 h after staged reperfusion. Management focused on minimizing clamp time, optimizing cardiac output with vasoactive support, correcting blood loss to maintain sufficient oxygen delivery, and gradually releasing the aortic and iliac artery clamps. Over 9 h of surgery, the patient received 510 mL of PRBC, 570 mL of FFP, 40 mL of platelets, and 320 mL of salvaged blood. Total blood loss was 750 mL, and urine output was 85 mL.

### 2.2. Postoperative Course

The patient remained intubated and was transferred to the PICU for invasive blood pressure monitoring, inotropic support, mechanical ventilation, and serial arterial blood gases. Vent settings were as follows: synchronized intermittent mandatory ventilation (SIMV), respiratory rate: 24, tidal volume: 90, positive end‐expiratory pressure (PEEP): 5, pressure support: 10 cmH_2_O, and fraction of inspired oxygen: 0.25. Postoperative goals were systolic blood pressure (SBP) of 70–80 and MAP > 55 mmHg, which were achieved with norepinephrine, epinephrine, and milrinone. Lower extremity Doppler examinations were performed for vascular monitoring. Lactate was measured every 6 h for 4 days to ensure appropriate renal clearance and normalized to 1.22 mmol/L at 12 h postoperatively. Cefazolin prophylaxis was continued for 72 h. Histopathologic examination demonstrated degenerative changes of the aortic media consistent with TSC‐related aneurysmal disease.

The patient remained sedated for 24 h per the intensivist. On Postoperative Day (POD) 1, sedation was maintained with dexmedetomidine 1.2 mcg/kg/hr, fentanyl 2 mcg/kg/hr, and midazolam 0.15 mg/kg/hr. Epinephrine and norepinephrine were discontinued, while milrinone continued at 0.5–0.75 mcg/kg/hr to support systolic and diastolic function and reduce systemic vascular resistance. SBP remained 70–80 mmHg with MAP 50–60 mmHg. Urine output averaged 1.2 mL/kg/hr. Repeat TTE revealed an improvement in EF to 43.7% (Figure 4) from 33.3%.

**FIGURE 4:**
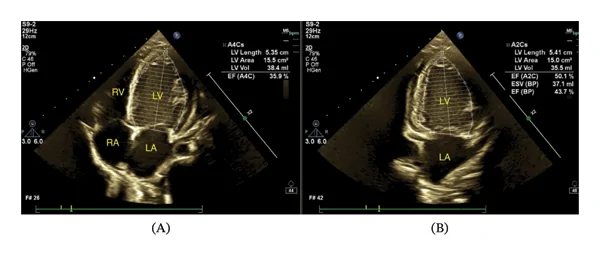
Postoperative Day 1 transthoracic echocardiogram in the apical four‐chamber (A) and two‐chamber (B) views demonstrating an improved ejection fraction (EF) of 43.7% by automated Simpson’s biplane tracing (BP). The left ventricular (LV) area in the four‐chamber view measured 15.5 cm^2^, compared with 17.2 cm^2^ preoperatively.

On POD 2, the patient was extubated to Vapotherm 10 L/min and FiO2 40%. Immediately following extubation, the patient had marked stridor, which improved with racemic epinephrine. Respiratory support was weaned to 2 L/min via nasal cannula over 24 h. Fentanyl provided sufficient analgesia and was transitioned to acetaminophen on POD 2 without issue. Milrinone was discontinued on POD 5. The patient was transferred to the ward on POD 6 and regained most of his physical function by POD 7.

The patient remained stable until discharge on POD 15. The prolonged hospitalization primarily reflected optimization of medical care before leaving Hawaii, including cross‐tapering vigabatrin to phenobarbital (which was available in their country) and coordinating follow‐up with hematology/oncology, neurosurgery, neurology, vascular surgery, and cardiology in 1 year. Annual MRA surveillance was planned, with graft replacement anticipated in 5–8 years as the patient grows.

At 1‐month telephone follow‐up, the patient had returned to his home country and remained stable without seizure recurrence. Return to Hawaii for surveillance imaging was delayed due to logistical constraints, with imaging planned approximately 2 years after surgery. No additional clinical information is available at this time.

## 3. Discussion

TSC‐associated AAA has been described in toddlers [8], but reports are limited and generally involve patients without significant cardiac dysfunction. Most pediatric AAAs require open repair because endovascular approaches are limited by a small vessel diameter, device availability, and the inability of current grafts to accommodate growth [9]. Although TEE is widely used during adult open AAA repair to assess ventricular filling and contractility [10], its use in pediatric repair is uncommon. There is no single published anesthetic protocol for pediatric open AAA repair, and TEE is rarely used unless cardiac dysfunction is present. Therefore, the authors employed TEE‐guided, goal‐directed fluid administration in accordance with pediatric anesthesia and heart failure guidelines.

Preoperative optimization included seizure control with vigabatrin and levetiracetam. As second‐generation antiepileptic drugs, they do not induce hepatic enzymes and are subject to fewer drug interactions [11]. Vigabatrin may potentiate the effects of GABAergic anesthetics such as propofol and should be titrated to effect. Antiepileptic therapy should generally be continued perioperatively because abrupt discontinuation can precipitate seizures.

A major perioperative goal during open AAA repair is to minimize cardiac stress, especially in the presence of impaired systolic function. Aortic cross‐clamping markedly increases afterload proximal to the clamp, placing significant stress on an already failing dilated LV [12]. This sudden increase in afterload may reduce stroke volume and precipitate ventricular decompensation. Interrupting blood flow to the lower extremities also alters venous return, causing a relative central hypovolemia as blood pools distal to the clamp. Addressing this concomitant increased afterload and reduced preload in a patient with reduced contractility requires careful volume administration to avoid both underperfusion and volume overload.

In the setting of dilated cardiomyopathy, continuous TEE provides information beyond conventional pressure‐based monitoring, allowing for real‐time assessment of LV filling, contractility, and response to interventions. TEE can detect deterioration in contractility or wall motion abnormalities earlier than CVP or EKG, respectively. In the LV short‐axis view, calculating the LV fractional area of contraction or using the simpler “eyeballing method” can provide information on contractility and the need for inotropes. This view can also be used to assess heart shape and chamber filling, providing a trend on preload and end‐diastolic volume [13]. Switching to the mid‐esophageal 4‐chamber view provides information on the right atrium and right ventricle, which can differentiate a preload deficit due to hypovolemia (small, underfilled right heart chambers) or right‐sided heart failure (severely dilated, hypokinetic, and pushing the interventricular septum into the LV) [14]. Hypotension with adequate LV filling and contractility suggests a vasodilatory or low systemic vascular resistance component; thus, titrating vasopressor support may be appropriate. Globally depressed contractility, especially after significant blood transfusion, should prompt correction of measured ionized calcium.

Massive transfusion was anticipated during open AAA repair [15]. Transfusion was guided by serial point‐of‐care hematologic and metabolic testing, blood loss, hemodynamics, and TEE. The authors utilized a balanced, weight‐based strategy, administering PRBCs and FFP in 20 mL/kg aliquots and platelets in 5–10 mL/kg aliquots during ongoing blood loss. This approach balanced oxygen‐carrying capacity and coagulation support while limiting excessive volume in the setting of LV dysfunction. When available, viscoelastic testing such as thromboelastography or rotational thromboelastometry (TEG/ROTEM) may further individualize transfusion during high‐risk surgery and in pediatric trauma [16], with cell salvage as a rescue mechanism.

Renal protection and metabolic management are also critical considerations during clamping. Aortic cross‐clamping at the infrarenal level can reduce renal perfusion by up to 45%, and the sudden reestablishment of blood flow upon unclamping can trigger an ischemia‐reperfusion event [17]. Mannitol is commonly used during aortic cross‐clamping because it creates an osmotic gradient that maintains tubular flow even during periods of reduced renal perfusion. Perioperatively, its proposed renal‐protective mechanisms include maintaining glomerular filtration pressure, preventing tubular obstruction, scavenging hydroxyl free radicals, and preventing tubular cellular swelling [18]. In practice, however, evidence for mannitol’s benefits is mixed, with studies showing possible protective effects and others demonstrating no difference for infrarenal patients. This patient’s preoperative renal function of BUN 20, creatinine 0.38, and GFR of 96 (using the Bedside Schwartz 2009 equation for pediatrics) was relatively similar to his postoperative values of BUN 16, creatinine 0.40, and GFR 91 and continued to improve.

During unclamping, reperfusion of the lower extremities can release lactic acid, potassium, free radicals, cytokines, and myocardial depressant factors, which may result in tissue damage, direct myocardial suppression, and profound peripheral vasodilation [19]. To reduce reperfusion injury, blood flow should be gradually restored during unclamping. This allows for slow systemic flushing of lactate and potassium, limiting the explosive free radical burst and preventing a massive drop in blood pressure [20]. As blood pools distally during clamping, this creates a relative hypovolemia that exacerbates unclamping hypotension. Volume loading is the traditional approach to mitigating unclamping hypotension, but prophylactic vasopressor support can be initiated to achieve tighter hemodynamic control. Our patient’s MAP decreased transiently below 50 mmHg after unclamping but quickly stabilized with vasopressors and gradual clamp release. EKG and TEE demonstrated stable cardiac function, suggesting that the patient tolerated unclamping without myocardial decompensation. To reduce the risk of thromboembolism from blood stasis, heparin is administered prior to aortic cross‐clamping, with additional doses administered for prolonged clamp times.

Near‐infrared spectroscopy (NIRS) would have been a reasonable adjunct to invasive monitoring to distinguish cerebral or renal hypoperfusion during this surgery [21]. While it is a low‐risk monitor, there is insufficient evidence to support or reject its clinical use. A systematic review of 25 randomized controlled trials found no significant effect of NIRS‐guided care on mortality or morbidity [22]. Measurements are also affected by technical (device variability) and physiological confounders (skull/tissue density and skin color), which may drive unnecessary interventions such as volume loading, transfusion, or pressor escalation. The authors believed that TEE, hemodynamic monitoring, and intraoperative labs throughout surgery were appropriate measures for monitoring end‐organ perfusion.

Regarding the postoperative course, an epidural or abdominal wall block could be incorporated to minimize opioid use. Epidural analgesia is the Society for Vascular Surgery guideline‐preferred technique for open AAA repair, as it has been shown to reduce initial postoperative pain scores, ventilation duration, postoperative respiratory failure, gastrointestinal bleeding, and ICU length of stay [23]. Finally, the decision for delayed extubation was made due to the patient’s large intraoperative blood loss and transfusion, ongoing vasopressor requirement, and lactic acidosis. However, delayed extubation following open aortic surgery has been associated with a prolonged length of stay and pulmonary complications, which should be taken into account for future graft replacement surgery [24].

This case highlights key perioperative considerations for open AAA repair in toddlers with dilated cardiomyopathy. Continuous TEE monitoring can help guide inotropic support and volume status. Early preparation for transfusion is essential due to the high risk of hemorrhage. Physiologic changes during cross‐clamping and reperfusion must be anticipated to prevent worsening ventricular dysfunction and metabolic acidosis. Finally, lifelong surveillance is necessary to monitor for aneurysm recurrence and eventual need for graft replacement.

## Funding

No funding was received for this manuscript.

## Disclosure

This report adheres to the CARE guidelines for case reports.

This report adheres to the applicable Enhancing the Quality and Transparency of Health Research (EQUATOR) guidelines.

Written authorization under the Health Insurance Portability and Accountability Act was obtained from the patient’s mother.

## Ethics Statement

Institutional ethical approval was not required for this single‐patient case report.

## Consent

Written informed consent was obtained from the patient’s mother for publication of this case report and any accompanying images.

## Conflicts of Interest

The authors declare no conflicts of interest.

## Supporting Information

Additional supporting information can be found online in the Supporting Information section.

## Supporting information


**Supporting Information** CARE‐checklist‐complete.

## Data Availability

The data that support the findings of this study are available on request from the corresponding author. The data are not publicly available due to privacy or ethical restrictions.
