# Supplementary material for: Perioperative Management of a Toddler With Tuberous Sclerosis and Dilated Cardiomyopathy Undergoing Open Abdominal Aortic Aneurysm Repair: A Case Report
Source: Case Rep Anesthesiol. 2026 Oct 16;2026:9513720. doi: 10.1155/cria/9513720 (PMC13674003; doi:10.1155/cria/9513720)
Supplement: Supplementary file 1 [file CRIA-2026-9513720-s001.pdf]

CARE case report guidelines

CARE Checklist of information to include when writing a case report

(cc) BY-NC-ND

Topic	Item	Checklist item description	Reported on Line
Title	1	The diagnosis or intervention of primary focus followed by the words "case report"	1-3 / page 1
Key Words	2	2 to 5 key words that identify diagnoses or interventions in this case report, including "case report"	15-16 / 1
Abstract (no references)	3a	Introduction: What is unique about this case and what does it add to the scientific literature?	2-4 / 2
	3b	Main symptoms and/or important clinical findings	5-6 / 2
	3c	The main diagnoses, therapeutic interventions, and outcomes	6-10 / 2
	3d	Conclusion—What is the main "take-away" lesson(s) from this case?	8-10 / 2
Introduction	4	One or two paragraphs summarizing why this case is unique (**may include references**)	2-17 / 3
Patient Information	5a	De-identified patient specific information.	2-3 / 4
	5b	Primary concerns and symptoms of the patient.	3-4 / 4
	5c	Medical, family, and psycho-social history including relevant genetic information	2-3 / 4
	5d	Relevant past interventions with outcomes	6 / 4
Clinical Findings	6	Describe significant physical examination (PE) and important clinical findings.	4-9 / 4
Timeline	7	Historical and current information from this episode of care organized as a timeline	2-39, 1-34 / 4-5
Diagnostic Assessment	8a	Diagnostic testing (such as PE, laboratory testing, imaging, surveys).	4-8 / 4
	8b	Diagnostic challenges (such as access to testing, financial, or cultural)	2 / 4
	8c	Diagnosis (including other diagnoses considered)	2-4, 31-32 / 4, 5
	8d	Prognosis (such as staging in oncology) where applicable	33-34 / 5
Therapeutic Intervention	9a	Types of therapeutic intervention (such as pharmacologic, surgical, preventive, self-care)	14-39, 1-21 / 4, 5
	9b	Administration of therapeutic intervention (such as dosage, strength, duration)	17-39, 1-21 / 4, 5
	9c	Changes in therapeutic intervention (with rationale)	21-24 / 4
Follow-up and Outcomes	10a	Clinician and patient-assessed outcomes (if available)	33-34 / 5
	10b	Important follow-up diagnostic and other test results	26-31 / 5
	10c	Intervention adherence and tolerability (How was this assessed?)	26-30 / 5
	10d	Adverse and unanticipated events	38-39, 13-15 / 4, 5
Discussion	11a	A scientific discussion of the strengths AND limitations associated with this case report	2-5 / 6
	11b	Discussion of the relevant medical literature **with references**.	2-27 / 6
	11c	The scientific rationale for any conclusions (including assessment of possible causes)	2-27 / 6
	11d	The primary "take-away" lessons of this case report (without references) in a one paragraph conclusion	30-35 / 6
Patient Perspective	12	The patient should share their perspective in one to two paragraphs on the treatment(s) they received	33-34 / 5
Informed Consent	13	Did the patient give informed consent? Please provide if requested	Yes ☒ No ☐
